# Possible Involvement of Locus-Specific Methylation on Expression Regulation of *LEAFY* Homologous Gene (*CiLFY*) during Precocious Trifoliate Orange Phase Change Process

**DOI:** 10.1371/journal.pone.0088558

**Published:** 2014-02-11

**Authors:** Jin-Zhi Zhang, Li Mei, Rong Liu, Muhammad Rehman Gul Khan, Chun-Gen Hu

**Affiliations:** Key Laboratory of Horticultural Plant Biology (Ministry of Education), College of Horticulture and Forestry Science, Huazhong Agricultural University, Wuhan, Hubei Province, China; Wuhan University, China

## Abstract

DNA methylation plays an essential role in regulating plant development. Here, we described an early flowering trifoliate orange (precocious trifoliate orange, *Poncirus trifoliata* L. Raf) was treated with 5-azacytidine and displayed a number of phenotypic and developmental abnormalities. These observations suggested that DNA methylation might play an important role in regulating many developmental pathways including early flowering trait, and then the expression level of five key or integrated citrus flowering genes were analyzed. Our results showed that *FLOWERING LOCUS T* (*CiFT*) relative expression level was increased with the increasing concentrations of 5-AzaC. However, *LEAFY* (*CiLFY*), *APETELA1* (*CiAP1*), *TERMINAL FLOWER1* (*CiTFL1*), and *FLOWERING LOCUS C* (*CiFLC*) showed highest relative expression levels at 250 µΜ treatment, while decreased sharply at higher concentrations. In order to further confirm DNA methylation affects the expression of these genes, their full-length sequences were isolated by genome-walker method, and then was analyzed by using bioinformatics tools. However, only one locus-specific methylation site was observed in *CiLFY* sequence. Therefore, DNA methylation level of the *CiLFY* was investigated both at juvenile and adult stages of precocious trifoliate orange by bisulfate sequencing PCR; it has been shown that the level of DNA methylation was altered during phase change. In addition, spatial and temporal expression patterns of *CiLFY* promoter and a series of 5′ deletions were investigated by driving the expression of a β-glucuronidase reporter gene in *Arabidopsis*. Exogenous GA_3_ treatment on transgenic *Arabidopsis* revealed that GA_3_ might be involved in the developmental regulation of *CiLFY* during flowering process of precocious trifoliate orange. These results provided insights into the molecular regulation of *CiLFY* gene expression, which would be helpful for studying citrus flowering.

## Introduction

A large proportion of many eukaryote genomes are variably methylated during the lifetime of the organism [1]. Previously, it has been reported that DNA methylation involved in X-chromosome inactivation [2], the silencing of transposons [3], multicopy genes, and genomic imprinting [4]. DNA methylation may be divided into two types. The first, known as maintenance methylation, maintains the original methylation pattern in the new strand of DNA upon replication. In the second, known as *de novo* methylation, unmethylated cytosines are methylated [5], [6]. DNA methylation is an epigenetic mark mediated by the addition of a methyl group to cytosine in DNA, but methyl groups can also be removed from DNA-by-DNA demethylation [7]. In both plants and animals, methylation involves the addition of a methyl group to a carbon at fifth position (C5) of the pyrimidine ring of cytosine in CpG dinucleotides (CpG islands) [8]. DNA methyltransferases then transfer a methyl group from S-adenosyl-L-methionine to cytosines in CpG dinucleotides [9]. However, high amounts of 5-methylcytosine found in some plant species suggested that methylation is not restricted to the CG sequence context and led to the discovery that cytosine is also methylated in CNG (*N* = A, T, G, or C) and, less abundantly, in CHH (*H* = A, T, or G) sequences [6], [10]. In plants, DNA methylation is more common in CpG islands, characteristic of transposons, contributing to the cytosine methylation increased levels, especially due to the high presence of these elements in plant genomes. These CpG islands are located in gene regulatory regions, aberrant methylation of CpG islands is characterized by transcriptional inactivation and subsequent loss of function of the gene regulated in this fashion without structural modifications [11], [12].

DNA methylation, one of the most abundant epigenetic modifications in higher plants, plays an important role in regulating developmental processes such as homeotic transformations in floral organs and altered flowering time [13], [14]. It is reported that *FWA*, *FERTILIZATION-INDEPENDENT SEED 2* (*FIS2*), and *MEDEA* (*MEA*) are controlled by genomic imprinting of DNA methylation [15]–[17]. *FWA* is involved in flowering time [18], [19] while *FIS2* and *MEA* regulate endosperm development [20]. The *FWA* promoter contains two pairs of transposon-associated tandem repeats that give rise to siRNAs [21], these are sufficient to induce DNA methylation and silence *FWA* expression, which is the default state [22], [23]. The induction of flowering by DNA demethylation was first reported for the low-temperature requiring ecotypes mutants of *Arabidopsis* [17], [24]. These data suggest that DNA methylation in plants regulates the expression of genes with important roles in morphogenesis and development, including molecular mechanism of flowering. However, the regulatory mechanism of woody plants by DNA methylation in relation to physiological events remains unclear so far.

The development of plants is characterized by juvenile and adult phases. After a relatively short juvenile phase, annual plants progress to the adult phase, during which flowering occurs. By contrast, perennial plants have a much longer juvenile phase, in some cases persisting for decades, which poses a major obstacle to the genetic improvement. Much less is known about the regulation of flowering developmental process in perennial species with particular growth habits [25]–[27], most information about the process regulation comes from studies in model plants. Therefore, an understanding of the genetic mechanisms underlying the flowering event is important for genetic improvement in perennial plants. Citrus is the most economically important fruit crop in the world. Its flowering has been the subject of ongoing investigation for many years [28]–[30]. Recently, several citrus homologs genes such as *LEAFY* (*LFY*), *APETELA1* (*AP1*), *TERMINAL FLOWER* (*TFL1*), *FLOWERING LOCUS T* (*FT*), *APETELA3* (*AP3*), *WUSCHEL* (*WUS*), and *FLOWERING LOCUS C* (*FLC*) can readily be isolated by exploiting the functional and sequence conservation of flowering genes among flowering plants [27], [29]–[33]. Molecular cloning and functional analyses of *LFY* have indicated that *LFY* may be a central regulator of the flowering regulatory network because the *LFY* gene is controlled by the autonomous, thermosensory, and gibberellin pathways in *Arabidopsis* [34]. The *LFY* protein is necessary and sufficient for the vital switch from vegetative to reproductive development in flowering plants [35]. Over-expression of *LFY* in transgenic plants can induce early flowering in annuals and perennials [36], [37]. However, to our knowledge, there are no reports of *LFY* methylation level during the phase transition in annual and woody plants.

Precocious trifoliate orange with a short juvenile phase derived from trifoliate orange (*Poncirus trifoliata* L. Raf), twenty percent of the seedlings germinated from the seeds flowered first in the next year after germination [38]. Thus, precocious trifoliate orange provides good material for studying the molecular mechanism of flower formation in woody plants. 5-Azacytidine (5-AzaC) is a pyrimidine nucleoside analog of cytidine that undergoes incorporation into DNA and blocks DNA methyltransferase leading to hypomethylation and potentially beneficial re-expression of abnormally silenced genes, reducing the overall level of DNA methylation in chromatin [39]. In annual plants, phenotypic changes induced by 5-AzaC have been reported in *Arabidopsis* [24]. Flowering time is the most recurrent and studied change; this process is accelerated in plants treated with 5-AzaC, which flowered earlier as compared with the control plants in *Linum usitatissimum* [40], *Perilla frutescens* [14], *Pharbitis nil* [41], and *Silene armeria* [42]. However, relatively few reports are available about woody plants. Here, we have reported that 5-AzaC applied to precocious trifoliate orange induced flowering genes expression, suggesting the involvement of DNA demethylation in the flowering process of precocious trifoliate orange. In addition, we have reported on *CiLFY* expression feature in the flowering transition stage, cloning, structural and functional analysis, and DNA methylation level during the phase transition of precocious trifoliate orange.

## Materials and Methods

### Plant material

Adult and juvenile precocious trifoliate orange samples were collected from the experiment fields of the National Citrus Breeding Center (30°28′ N, 114°21′ E, 30 m) at Huazhong Agricultural University. Apex bud and the following five buds (the major node position for flower formation) from spring flushes were sampled every two months in the year after bud swelling, the adult trees were 3- to 5-year-old. The juvenile material was seedlings germinated from the seeds of the adult mother plants. Because the embryo originates from a nucellar cell in trifoliate orange, the seedlings have the same genetic background as the mother plants. The seeds of precocious trifoliate orange were planted in 20-cm pots containing potting mix of commercial medium and perlite at a ratio of 3∶1; the juvenile trees were watered regularly with nutrient solution. Shoot apical meristems of the juvenile tree were collected in March, June, September, and December. For spatial expression analysis of *CiLFY* gene, several plant organs from juvenile and adult stages (lateral buds, apex buds, stems, leaves, flowers at full bloom, and whole fruits at 30 days after flowering) were sampled and immediately frozen in liquid nitrogen and stored at −80°C until use. All materials were collected from three individual plants for RNA extraction.

### 5-Azacytidine treatment

5-Azacytidine (Sigma, Switzerland) solution was freshly prepared for each experiment in phosphate-buffered saline (PBS) at a concentration of 2.4 mg/ml (10 mM) and sterile filtered. After the seed coats of precocious trifoliate orange seed were peeled and sterilized, the embryos were imbibed at 23°C on filter paper soaked with fresh 5-AzaC solution (0, 250, 500, and 1000 µΜ). The seeds were transferred daily to new filter paper containing fresh 5-AzaC solution. After 15 days, the germinated seeds were planted in 20-cm pots containing potting mix of commercial medium and perlite at a ratio of 3∶1. The seedlings were watered regularly with nutrient solution and grown in test tubes in artificially lit growth cabinets under long days (16 h light and 8 h dark at 23°C) with fluorescent lights at a photosynthetic photon flux density of 200 µmol m-2s-1.

### Analysis of flowering related genes expression by Real-time PCR

Total RNA was extracted according to a previous protocol [30]. The expression levels of flowering related genes were investigated by using real-time PCR with SYBR green I chemistry (QIAGEN, Germany). Primers were designed with the Primer Express software and tested to ensure amplification of single discrete bands with no primer-dimers. Total RNA (3 mg) was treated with 3 units of DNase (Promega, USA) and then used in first-strand synthesis with an oligo (dT) primer (20-mer) and reverse transcriptase according to the manufacturer's instructions. For real-time PCR, an amount of cDNA corresponding to 25 ng of input RNA was used in each reaction. Real-time PCR was performed on the LightCycler™ 480 System (Roche Applied Science, Mannheim, Germany) using *β-actin* as endogenous control. Briefly, the primers were diluted in the SYBER GREEN PCR Master Mix and 20 µl of the reaction mix was added to each well. Reactions were performed by an initial incubation at 50°C for 2 min and at 95°C for 1 min, and then cycled at 95°C for 15 s and 60°C for 1 min for 40 cycles. Data were evaluated by calibrator-normalized relative quantification with efficiency correction using the LightCycler™ 480 software version 1.5 (Roche Applied Science, Mannheim, Germany) and normalized to expression of *β-actin*. Real-time quantitative PCR was performed in four replicates for each sample, and data were indicated as means ± SD (n = 4). Three biologic repeats were assayed for each sample in this study, giving similar trends. Data from one biologic repeat were presented.

### The promoter of flowering related genes isolation and bioinformatic analysis

High quality DNA was extracted according to Cheng et al [43]. Four Genome-Walker libraries were constructed by using the Genome-Walker kit (Clontech, USA) according to the manufacturer's manual. Genomic DNA was digested with the restriction enzymes *Dra* I, *EcoR* V, *Hpa* I and *Sca* I. Following digestion, each pool of DNA fragments was ligated to the Genome-Walker Adaptor. The upstream genomic region was amplified from each library using two nested adaptor primers and two nested gene-specific primers. The primary PCR amplification was used for the outer adaptor primer (AP1) provided in the kit and the outer gene-specific primer. The primary PCR mixture was then diluted to 50 folds, and 1 µl of the diluted primary PCR mixture was used as a template for the secondary or “nested” PCR amplification by using the nested adaptor primer (AP2) and the nested gene specific primer. The *CiLFY* promoter and gene have been deposited in GenBank under Accession no. FJ238533 and AY338976, respectively.

### Bisulfite DNA sequencing

Bisulfite treatment was performed as described with some modifications [44]. Genomic DNA (3 µg) was digested with *EcoR* I and *EcoR* V and purified. DNA (0.5 to 2.0 µg) in 18 µl water was denatured at 95°C for 5 min. After quenched on ice, 3 M NaOH (2 µl) was added and incubated at 37°C for 15 min. The bisulfite solution was prepared by dissolving 5.1 g of sodium bisulfite (Sigma, S-9000) in 8 ml of water with slow stirring to avoid aeration. The pH was adjusted to 5.1 with freshly prepared 10 M NaOH. Then, 330 µl of 20 mM hydroquinone (Sigma, H-9003) was added and the volume was adjusted to 10 ml with water. Pre-warmed bisulfite solution was added to the DNA solution, mix gently, and overlaid with mineral oil. The bisulfite conversion was conducted by using 5 cycles of 95°C for 5 min, 55°C for 3 h. After bisulfite treatment, DNA was desalted with Wizard® DNA clean-up system (Promega, USA) following the manufacturer's instructions and recovered in 40 µl of water. 3 M NaOH (4.5 µl) was added to the DNA solution and incubated at 37°C for 15 min. DNA was then recovered by ethanol precipitation with glycogen carriers and 5 M NH_4_OAc and dissolved in 25 µl TE buffer (10 mM Tris–HCl, 1 mM EDTA, pH 8.0), and 1 µl aliquot of the bisulfite treated DNA was used for each 20 µl volume of touchdown PCR reaction with three specific sets of primers. Reactions were performed by 94°C/0.5 min×1 cycle; 94°C/0.5 min, 60°C to 50°C/45 s, 72°C/1.5 min, −2°C/2 cycles; 94°C/0.5 min, 50°C/45 s, 72°C/1.5 min, ×30 cycles; and 72°C for 10 min for total 40 cycles. Products were cloned and seven individual clones from each tissue were sequenced. The process was repeated three times using biologically independent samples; each biologically sample was collected from three individual plants for DNA extraction in this study.

### Vector construction and *Arabidopsis* transformation

Full-length promoter (Del 0) and a series of 5′-deletions fragments (Del 1-Del 5) were cloned into the pCAMBIA1391z vector, respectively. Then these vectors were transformed into *Agrobacterium tumefaciens* strain EH105. *Arabidopsis* ecotype Columbia (Col) plants were transformed by the floral dip method [45]. T_0_ generation seeds were sterilized and germinated on the 1/2 MS [46] solid medium plates containing 25 mg/L hygromycin B (Roche, Germany) as a selective agent in the long-day conditions (16 h light and 8 h dark). Seven days later, positive T_1_ seedlings were transplanted to soil to grow at the same photoperiod. One mg/mL GA_3_ (Sigma, USA) was added to T_3_ seedlings plated on 1/2 MS medium to obtain 100 µM working concentration [47]. Application of exogenous GA_3_ after transplanting plants on soil growth was achieved by spraying soil-grown plants twice weekly with a solution of 100 µM GA_3_ and 0.02% Tween-20 (Bio-Rad). Samples were collected the day after spaying.

### Histochemical localization and fluorometric measurement of GUS activity

GUS staining was carried out as described by Jefferson with some modifications [48]. Various tissues from transgenic *Arabidopsis* were immersed in X-Gluc solution (1 mg/ml X-Gluc, 100 mM sodium phosphate buffer [pH 7.0], 10 mM EDTA, 1 mM potassium ferricyanide, 1 mM potassium ferrocyanide, 1% TritonX-100, 100 µg/mL chloramphenicol, 20% methanol) and incubated for 16–24 h at 37°C, and then immersed in 70% ethanol for 3 to 4 times.

For fluorometric measurement of GUS activity, plants were ground to a fine powder and then suspended in 1 ml GUS extraction buffer (50 mM sodium phosphate, pH 7.0; 0.1% Triton X-100; 10 mM β-mercaptoethanol; 10 mM EDTA and 0.1% sarcosyl (v/v)). The supernatant, after being centrifuged at 12,000 g for 20 minute at 4°C, was assayed for GUS activity with 4-methyl umbelliferyl glucuronide (Sigma) substrate using an F-4500 fluorescence spectrophotometer at the excitation/emission wavelengths of 365/455 nm. The protein concentrations were quantified according to Gallagher and the GUS enzyme activity was expressed as nmols of 4-methylumbelliferone produced per mg protein per minute [49]. In this study, all materials were collected from three individual transgenic lines for GUS staining and measurement of GUS activity.

## Results

### Characterization of plants by 5-Azacytidine treatment

**Figure 1:**
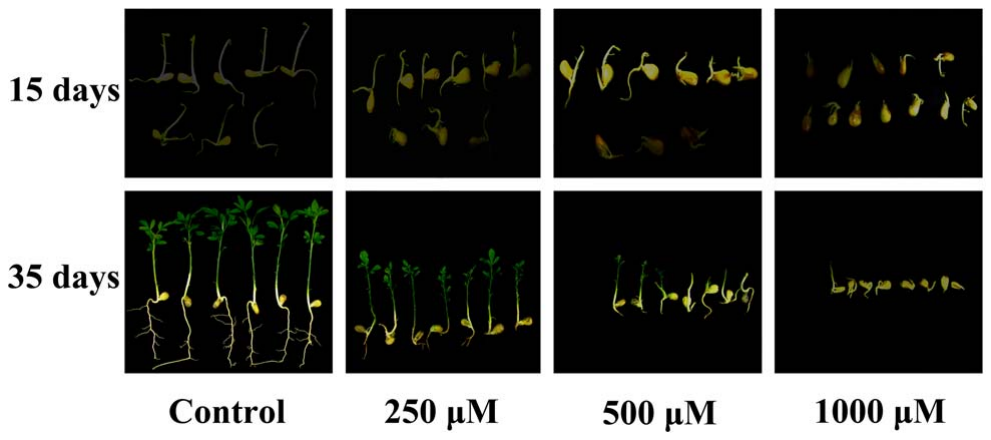
Phenotypic characteristics of different concentrations of 5-AzaC treated precocious trifoliate orange seeds grew for 15 and 35 days, control: treated with distilled water. After the seed coats of precocious trifoliate orange seed were peeled and sterilized, the embryos were imbibed at 23°C on filter paper soaked with fresh 5-AzaC solution (control, 250, 500, and 1000 µΜ). The seeds were transferred daily to new filter paper containing fresh 5-AzaC solution. After 15 days, the germinated seeds were planted; the seedlings were watered regularly with nutrient solution and grown in test tubes in artificially lit growth cabinets under long days (16 h light and 8 h dark at 23°C) with fluorescent lights at a photosynthetic photon flux density of 200 µmol m-2s-1. The treated seedlings were analyzed after the seedlings grew in the soil for 20 days.

Different concentrations of 5-AzaC were treated continuously precocious trifoliate orange seeds at 0, 250, 500, and 1000 µΜ for 15 days under dark conditions (Figure 1). The treated plants were distinguishable from the control plants (treated with distilled water), the increasing concentrations of 5-AzaC significantly retarded the seedling development especially the root development (Figure 1). For further investigations, these treated seedlings were transplanted to the soil. After 20 days of transplantation, it was noticed that the roots of seedlings treated with 250 µΜ 5-AzaC grew slowly as compared with the control plants (Figure 1). There were several aberrant leaves grown on seedlings. In addition, there was quite less number of vegetative growing buds, which then tends to decrease with the increase in time. However, seeds treated with 500 µΜ 5-AzaC concentration were showing apparently inhibited root and stem development of the seedlings (Figure 1). Shoot apices of some plants were died which then prevented the further elongation of the main stem. Most of the leaves exhibited by the seedlings were having aberrant phenotype. Moreover, the growth of 1000 µΜ 5-AzaC treated seedlings was nearly standstill compared with 20 days before condition (Figure 1). By summarizing, our results revealed that the plant height was reduced proportionally more with increasing 5-AzaC concentrations as compared with the untreated seedlings.

### Analysis of flowering related genes in 5-Azacytidine treated plants by Real-time PCR

**Figure 2:**
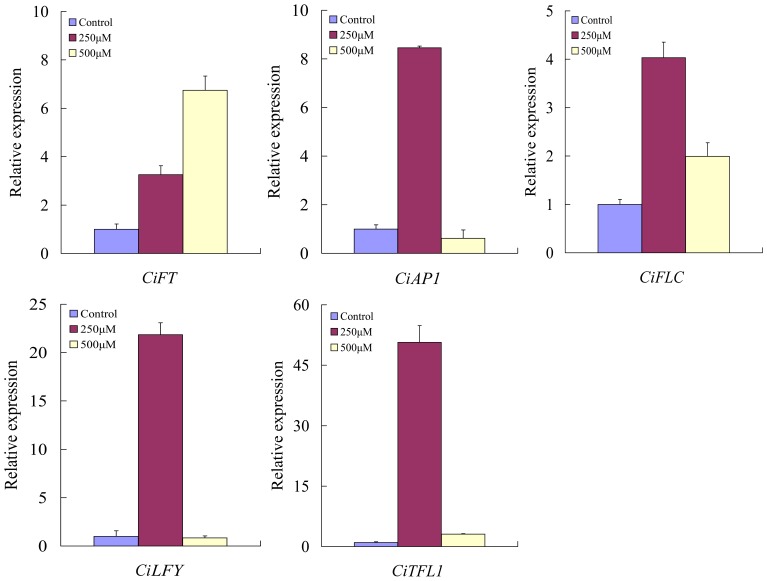
The relative expression analysis of*CiFT, CiAP1, CiFLC, CiLFY*, and *CiTFL1* under different 5-AzaC concentrations by real-time PCR. Total RNA was isolated from throughout the aerial part (including stem, leaf and shoot apical meistem) of 35 days after seed germination as shown in Figure 1. Data points represent mean values ± SE of at least four replicates for the relative expression, which were normalized by the amount of the *β-actin* control expression. The primers used for the analyses were listed in table S1

In order to further understand the involvement of DNA methylation in flowering process of precocious trifoliate orange, total RNA was isolated from throughout the aerial part (including stem, leaf and shoot apical meistem) of 35 days after seed germination with different 5-AzaC concentrations for analyzing some flowering related genes (Figure 2). Previous reports have revealed that *TFL1*, *FT*, *AP1*, *FLC* and *LFY* play a critical role during the early flowering process of model and woody plants. Therefore, the citrus homologous gene of these genes such as *CiTFL1, CiLFY, CiAP1, CiFLC, CiFT* were investigated under different 5-AzaC concentrations. Our findings exhibited that the *CiFT* expression level was increased with the increasing concentrations of 5-AzaC (from 250 to 500 µΜ). However, *CiTFL1, CiLFY, CiAP1*, and *CiFLC* showed highest relative expression levels in 250 µΜ treatment and followed by a sharp decrease in the relative expression in the seedlings treated with 500 µΜ concentration (Figure 2). These results revealed that demethylation treatment directly or indirectly influences the expression of the above genes during early flowering process of precocious trifoliate orange.

### Sequence characterization of flowering related genes promoter

CpG and CNG islands are the worthy targets for DNA methylation [6], [10]. To confirm DNA methylation affects the expression level of these flowering genes, we focused on specific DNA methylation site in these genes. Therefore, the promoter sequence (about 1.5 kb–2.0 Kb) of the *CiTFL1, CiLFY, CiAP1, CiFLC* and *CiFT* was isolated from precocious trifoliate orange. The 5′ upstream region and full-length gene of the above five genes were analyzed by using NSITE, TSSP, NSITEM and PromoterScan software. The results revealed the presence of common elements such as TATA box and CAAT box, the putative transcriptional start site and different binding motifs (circadian rhythms; light regulation) in these promoters. However, only one methylation site (CpG Island) was observed in *CiLFY* sequence with Methyl Primer Express v1.0 (Figure S1). The CpG island was located at the tail of 5′-UTR region and gene (from +609 to +1255 bp). The bioinformatic analysis of the region results as A%: 25.81%; T%: 21.10%; C%: 21.33%; G%:30.76%; C+G%: 52.09%; CG%: 4.8; A+T/C+G%: 0.92%, and sequence analysis by CyMATE was CHH% (*N* = A, T, G, or C): 47.8%; CHG% (*H* = A, T, or G): 26.7%; CG%: 25.6%. The result indicated that the *CiLFY* may be regulated directly by DNA methylation in precocious trifoliate orange.

In addition, bioinformatic analysis revealed that the putative transcription start site (A) was located at 746 nucleotides upstream to the start codon (ATG) of the *LFY* promoter consistent with 5′-RACE (rapid amplification of cDNA ends) experiment. A putative TATA box and a putative CAAT box, were located at the regions −25 (−)/−200 (−), −49 (−)/−90 (−), respectively. The two highly conserved motifs that were involved in the interaction with the RNA polymerase and in the regulation of gene transcription efficiency, respectively. Based on the PLACE software analysis, 394 distinct putative *cis*-regulatory elements were identified within the *CiLFY* promoter. Of the 394 *cis*-elements, 40 were singletons, and the remaining was assembled into 50 groups with occurrence ranging from 2 to 28 times (Table S2). There were a number of phytohormone responsive motifs such as auxin response factor binding motifs (ASF1MOTIFCAMV and AUXRETGA1GMGH3), gibberellin response motifs (PYRIMIDINEBOXOSRAMY1A), and ABA response motifs (EBOXBNNAPA and MYCCONSENSUSAT). Various putative elements in relation to light response were also abundant in *CiLFY* promoter, signifying that this promoter was probably subject to light regulation and involved in photoperiod pathway (Table S1). Interestingly, two flowering gene binding sites (*AGAMOUS-like 15* and *WUSCHEL* gene) were also identified in *CiLFY* promoter. These sites were potentially involved in flowering and apical meristem development; suggesting a regulatory relationship with the two genes in the *CiLFY* promoter. In addition, a number of potential regulatory motifs corresponding to known *cis*-regulatory signals of eukaryotic genes were also found, including low-temperature-responsive elements, circadian control factors, early responsive to dehydrations, enhancer elements and so on (Table S2).

### Analysis of the *CiLFY* expression at juvenile and adult stages by real-time PCR

**Figure 3:**
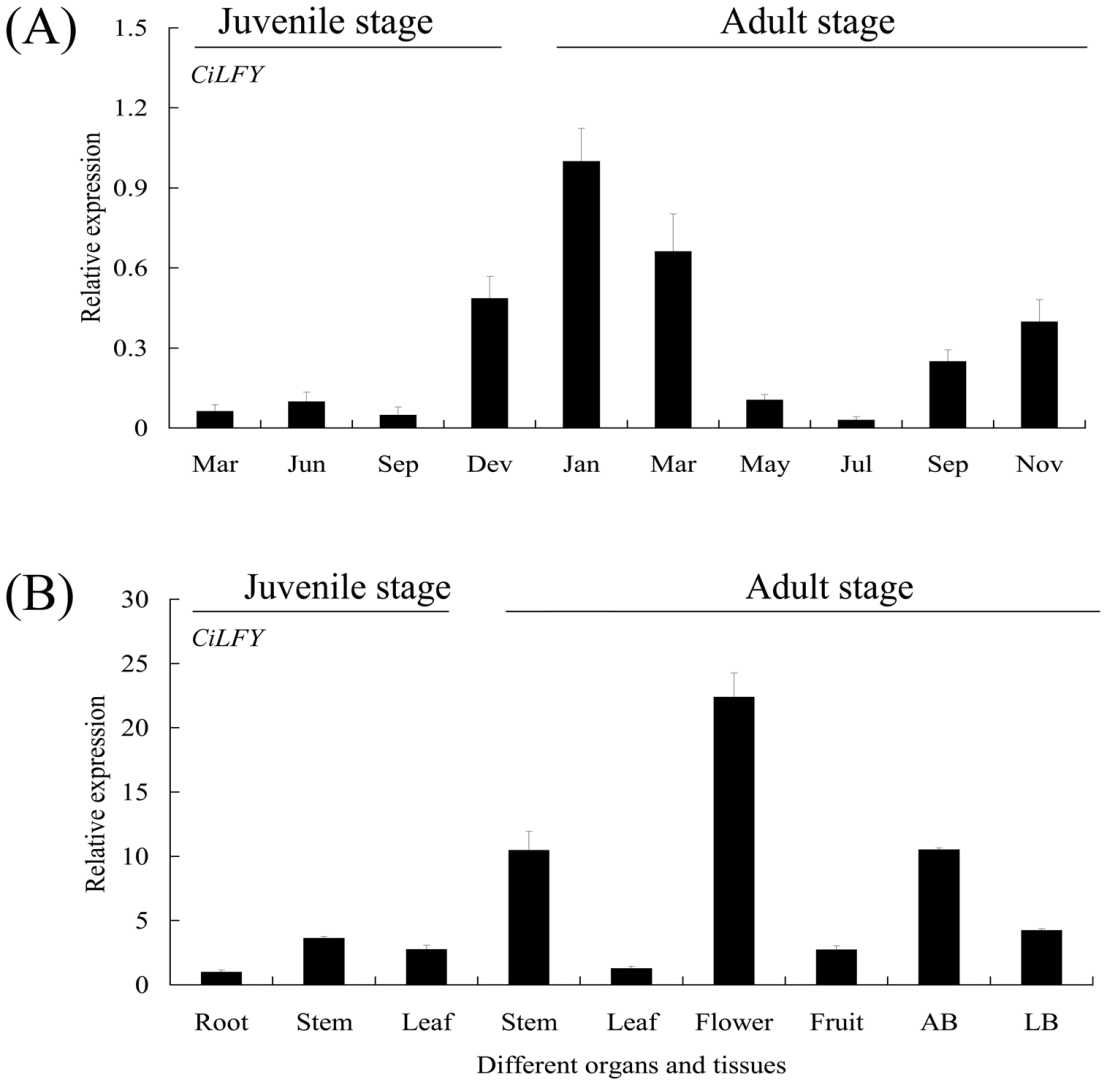
Relative quantities of *CiLFY* in various tissues and stages of precocious trifoliate orange. (A): Relative quantities of *CiLFY* at juvenile and adult stage of precocious trifoliate orange. (B): Relative quantities of *CiLFY* in various tissues of juvenile (leaves, roots and stems) and adult (leaves, roots, stems, flowers at full bloom, whole fruits at 30 days after flowering, apex bud and lateral bud) phase of precocious trifoliate orange. AB: apex bud, LB: lateral bud. Data points represent mean values ± SE of at least four replicates for the relative expression, which were normalized by the amount of the *β-actin* control expression. The primers used for the analyses were listed in table S1

A major characteristic of precocious trifoliate orange was that its juvenile phase was shortened to 1 to 2 years. The flower buds of precocious trifoliate orange cannot be recognized visibly by shape and size at early stages. For this reason, paraffin section was performed to identify flower development stage during the transition from the vegetative to reproductive stage. From November to January, some vegetative growth points were transformed into flower buds, it becomes broadened and flattened, forming floral apical meristem, finally give rise to flower meristem and flowers (March) [33]. To understand the relationship between phase change and the expression of *CiLFY* in precocious trifoliate orange, the expression level of *CiLFY* was investigated by real-time PCR (Figure 3). As a result, there was a fluctuation in the *CiLFY* expression level accompanying with the season shift and morphological change. The level of *CiLFY* was low during summer and autumn, it slowly climbed up after December and peaked in January, and maintain in high level in March, and then the mRNA level decreased rapidly (Figure 3A). In addition, high levels of *CiLFY* gene were reflected in the adult tissues as compared with juvenile tissue (Figure 3A). One possible explanation for this observation is that this gene may play an important role in inducing early flowering in precocious trifoliate orange and may be directly regulated by methylation.

On the other hand, the temporal expression of *CiLFY* was further examined by real-time PCR in juvenile (leaves, roots and stems) and adult (leaves, roots, stems, flowers, fruits, apex bud and lateral bud) different tissues (Figure 3B). Our results revealed that this gene have shown broad expression patterns, with the transcripts detected in all plant organs except adult roots. The expression levels were particularly high in juvenile stems and adult stems, flowers and apex buds (Figure 3B). As the apex buds are the major node position for flower formation, so this results suggested that the expression may be associated with floral development in the early flowering process of precocious trifoliate orange.

### Methylation status of *CiLFY* at juvenile and adult stages of precocious trifoliate orange

**Figure 4:**
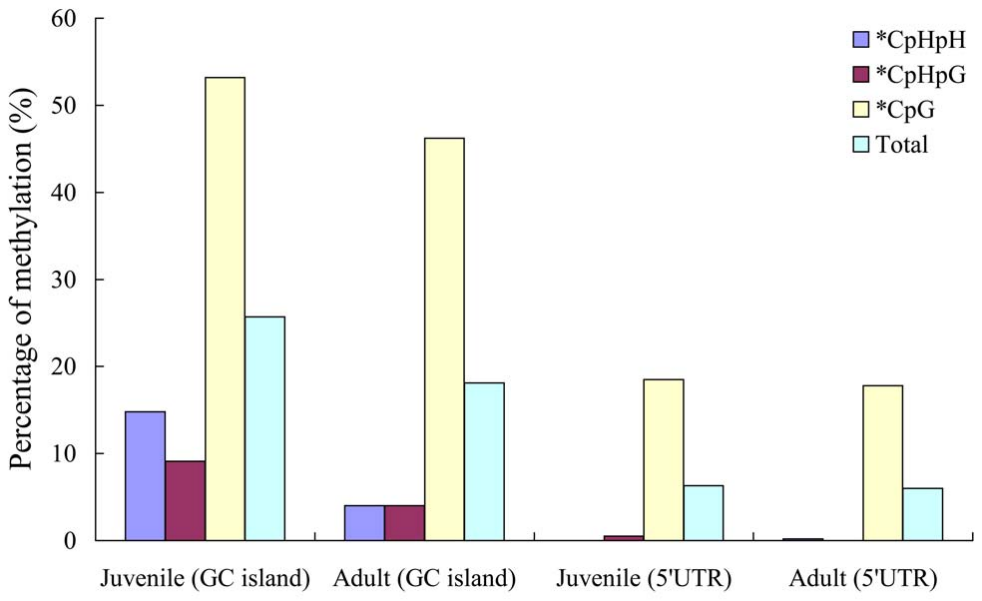
Statistical analysis of the cytosine methylation status in CpG island and 5′-UTR of *CiLFY* gene at juvenile and adult phase of precocious trifoliate orange.

Locus specific methylation analysis of the *CiLFY* CpG island was performed to uncover the relationship between the phase change of the precocious trifoliate orange and the methylation status (Figure 4). Observations revealed that the overall DNA methylation status of *CiLFY* was 25.7% and 18.1% at juvenile and adult stages, respectively. The proportion of three different methylation cytosine for juvenile stage was CG%: 52.3%; CHH%: 14.8%; CHG%: 9.1% and for adult stage was CG%:46.2%; CHH%: 4%; CHG%: 4%, respectively (Figure S1). These results indicated that the prominent methylation decrease during the transition from juvenile stage to adult stage in precocious trifoliate orange. DNA methylation occurs mainly at CG sites in mammals, but these often have been observed at CNG and CHH sites in plants [6], [10]. In this study, CHH and CNG were relative abundant in *CiLFY* 5′ UTR. Therefore, DNA methylation level of the *CiLFY* 5′ UTR was also analyzed. Interestingly, bisulfite genomic sequencing results indicated that the overall DNA methylation status of *CiLFY* 5′-UTR was 6.3% and 6.0% at juvenile and adult stages, respectively (Figure 4). The proportion of three different methylation cytosine was 18.5 and 17.8% (CG); 0.5 and 0% (CHH); and 0 and 0.2% (CHG) at juvenile and adult stages, respectively (Figure S1). Therefore, the demethylation of *CiLFY* might be the reason for the reflection of gene expression during flower initiation. The results also suggested that there may be an association of *CiLFY* demethylation to the phage transition in precocious trifoliate orange.

### Spatial and temporal expression patterns of *CiLFY* promoter in *Arabidopsis*

**Figure 5:**
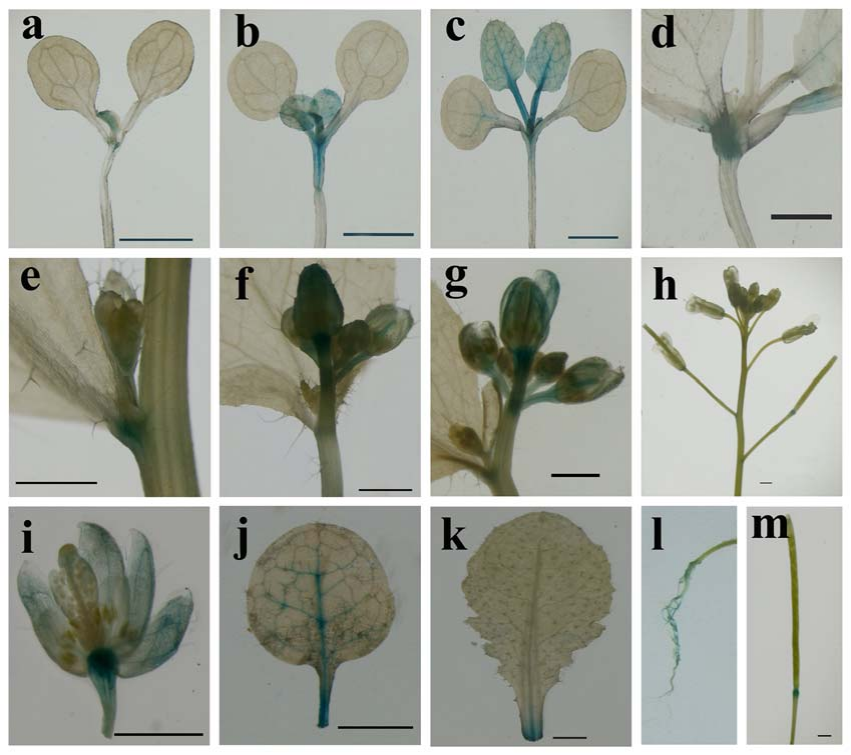
Histochemical localization of GUS activity in transgenic *Arabidopsis*. a–d: seedlings at 7, 10, 14 and 20 days, respectively; e: lateral flower bud just emerged; f: lateral flower bud; g: apex flower bud; h: young inflorescence; i: fully-opened flower; j: young leaves; k old leaves; l: roots from adult plants; m: fruit. bar: 1 mM.

To examine the tissue specificity of the *CiLFY* promoter, a GUS expression construct driven by full-length *CiLFY* promoter (1,641 bp) was introduced into *Arabidopsis* (Figure 5). Transgenic plants were confirmed for the presence of appropriate expression cassette by PCR. Overall, histochemical analysis have indicated that the GUS enzyme activity was detected in adult roots, young leaves, floral petals and sepals, but was not in the pistil, stamens, or seeds (Figure 5). At juvenile stage, GUS expression was first detected in 7-day-old seedlings, there was stronger GUS staining in the first two true leaves, while there was relatively weaker staining in the hypocotyls, but no staining was observed in cotyledon and radicles. Similar GUS activities were maintained in 14-day-old aerial parts (Figure 5). Histochemical assay at adult stage indicated that GUS staining was found in all floral buds (including apex and lateral). Detailed flower study has revealed that the sepals, peripheral petal, and stigmatic papillae exhibited very intense GUS expression (Figure 5), while anther locules and stamen filaments did not exhibit any GUS expression (Figure 5). The other aerial plant parts like stem, bracts and mature leaves did not exhibit any GUS expression. In fruit, GUS expression was only confined to fruit abscission zone. The GUS expression pattern was also corroborated with the pattern observed by real-time PCR (Figure 3B). All of these results indicated that the *CiLFY* promoter modulated precise transcriptional regulation of specific and developmental expression in transgenic *Arabidopsis*.

### Expression of promoter-GUS response to GA_3_ treatment in transgenic *Arabidopsis*

**Figure 6:**
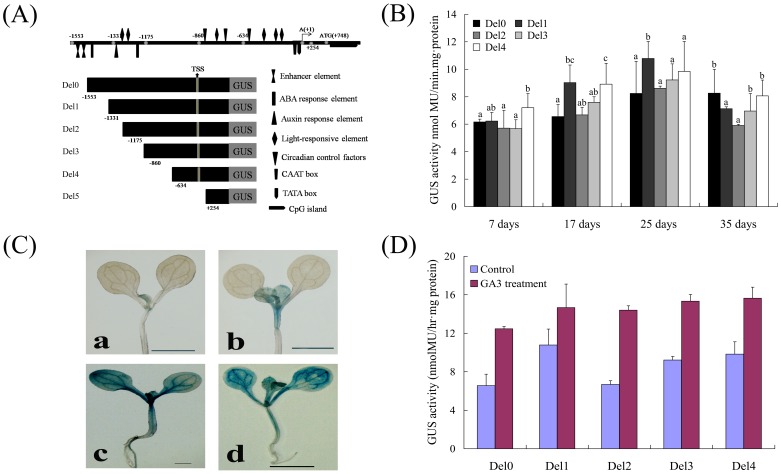
GUS activity of the *CiLFY* promoter in transgenic *Arabidopsis*. (A): *CiLFY* full-length promoter and deletion derivatives. Numbers inside the bars indicated the end position of each deletion, number 1 (+1) represents the first nucleotide on the 5′ side of the transcription start site (TSS). (B): Folds increase in the GUS activity for the transgenic plants with full-length and various truncated of the promoters. Three independent transgenic lines for each of the promoter fragments were used for the assay. Error bars represent standard error. C: Histochemical localization of GUS activity in under untreated (a and b) and 100 µM GA_3_ treated (c and d) transgenic *Arabidopsis*. D: Analysis of GUS activity from *CiLFY* deletion constructs in 17-old-days transformed *Arabidopsis*, Leaves were collected the day after spaying 100 µM GA_3_. Error bars represent standard error. The results from one representative experiment were shown herein, expressed as means and standard errors calculated using Microsoft Excel. The data were processed using one-way analysis of variance (ANOVA), and statistical differences were compared based on Student's t-test, taking P<0.05 as significant.

To gain further insight into the functional role of *CiLFY* promoter regions, a series of expression cassettes with truncated promoter fragments based on the organ/tissue-specific and hormones responsive region and a GUS reporter gene were generated (Figure 6A). In this study, five deletions were constructed and transformed into *Arabidopsis*. As a result, histochemical GUS staining on transgenic plant of deletion of promoter fragment have showed the same positional distribution as compared to that of the full-length promoter (Del 0), but with different expression intensity at different development stages. Del 1 showed stronger promoter activity as compared with Del 0 and Del 2 (Figure 6B), which possibly suggest negative regulating element (−1553 to −1331 bp) and positive element (−1331 to −1175 bp) in thses regions. We have noticed the presence of a “CACGTGMOTIF” motif in the promoter sequence between −1,433 and −1,439. This element was of our interest because of its behavior as a transcriptional repressor element, which then involved in regulation of transcription (Table S2). Meanwhile, a CCAATBOX1 element was also identified, which is a *cis*-enhance regulatory element essential for increasing the promoter activity. Del 3 showed almost the same activity intensity with Del 2, but Del 4 exhibited the strongest promoter activity amongst them (Figure 6B). It means that there may be a very strong negative element in the region (from −860 to −634 bp). Del 5 (from +254 to +768) did not show any activity, which may indicate the basic promoter element region between −634 and +1 as predicted by Matinspector. From the observations described above, it was therefore conceivable that there was almost no organ-specific located at the region from −1553 to −634 bp. However, there was an obvious intensity difference in terms of different constructs because of the presence of some enhancers and the inhibitors in *CiLFY* promoter region.

The gibberellin class of plant hormones has been implicated in the control of flowering in several species. Previous studies indicated that gibberellin promote flowering of *Arabidopsis* by activating the *LEAFY* promoter. In this study, there was one gibberellin inductive motif PYRIMIDINEBOXHVEPB1 (−177) in *CiLFY* promoter (Table S2). Therefore, the histochemical GUS activity assays were complemented by analyzing the GA_3_ induced expression of the GUS reporter gene in transgenic *Arabidopsis* harboring the different *CiLFY* promoter-GUS fusion constructs (Figure 6D). Quantitative assay exhibited the enhancement of the full-length promoter activity as compared with control at juvenile stage (Figure 6C). GUS staining was all over the seedlings including radicle, which was absent in the un-treatment. At adult stage, plants exhibited the same expression pattern in terms of flowering organs with un-treatment transgenic plants except significantly enhanced the expression of GUS. For progressive 5′ deletions, the expression of GUS have a sharp increase compared with un-treatment plants, while the expression of GUS gene did not show significant differences among different deletions (Figure 6D). These results suggested that gibberellins inductive motifs “PYRIMIDINEBOXHVEPB1” was essential for GA_3_ induction in citrus. The above results demonstrated that the promoter had a mechanism responding to gibberellin condition in transgenic *Arabidopsis* plants and this responding site was within the Del 4 region.

## Discussion

DNA methylation, one of the most abundant epigenetic modifications in higher plants and animals, plays an important role in regulating developmental processes. Recently in plants, epialleles of genes with ecological importance having effect on development, floral morphology, flowering time, seed pigmentation, and pathogens resistance, have been characterized [1], [13], [14], [41]. Although, early flowering induced by 5-AzaC treatment has reported in annual plants, we report here for the first time on the flower-induction by DNA demethylation in woody plants. There were some morphological abnormalities observed in precocious trifoliate orange by 5-AzaC treatment. The abnormal plants have decreased their stature, smaller leaves, leaves with margins curled toward the upper leaf surface, and reduced apical dominance. Therefore, we supposed that the developmental abnormalities were correlated with altered patterns of gene expression. Treatment of plant and animal cells with 5-AzaC resulted in the demethylation of DNA directly by incorporation of the analogue in place of cytosine during DNA replication [50] and indirectly by inhibiting of the action of methyltransferase [9]. Demethylation of DNA by 5-AzaC has been correlated with induction of transcription in a number of gene systems in plants [14], [50]. It has been reported that the reduced DNA methylation by 5-AzaC treatment in *Arabidopsis* was also resulted in abnormal plant development [42], [51]. There was no flowers observed after the application of different concentrations of 5-AzaC. There might be two possible explanations: at first, genetic regulation of floral induction in perennial species is much more complex than in annual plants, so demethylation treatment may cause the metabolic disorders in treated plants. There is evidence supporting the view that demethylation resulted in abnormal plant development. Secondly, the juvenile period of precocious trifoliate orange has been greatly reduced to 1–2 years as compared with the other citrus plants. Thus, the flowering induced by different treatments of 5-AzaC in precocious trifoliate orange was accompanied by only suppression of vegetative growth with no obvious changes in flowering time. Therefore, it is necessary to examine the effect of 5-AzaC in long-juvenile citrus plants which have members showing early flowering and late flowering in the future.

Vernalization-requiring late-flowering mutants *fca* and *fy* of *Arabidopsis* have induced early flowering by 5-AzaC treatment [24], 5-AzaC also induced flowering in the vernalization-requiring *Thlaspi arvense*, *Perilla frutescens* and potatoes [13], [24], [52]. These results indicated that the flowering genes may be up-regulated through the decrease DNA methylation in annual plants [53], [54]. Recently, it has been reported that the expression of the *FT* was statistically increased in the 5-AzaC-treated early flowering plants with respect to control plants in potato [13]. Previous studies also exhibited the higher transcription levels and involvement of *FT*, *AP1*, *TFL1*, *FLC* and *LFY* homologous genes during flower induction as well as floral induction, inflorescence development and flowering [32], [55]–[58]. These results indicated that these genes might have an important role in inducing early flowering and shortening the juvenile phase. Our results have reflected an increased relative expression level of *CiFT* with different 5-AzaC treatments. However, *CiTFL1, CiLFY, CiAP1,* and *CiFLC* showed highest relative expression levels at 250 µΜ concentration. In the past investigations, the transcript level of floral inhibitor *FLC* is down regulated by treatment with 5-AzaC in *Arabidopsis* [42], [52], , while *CiFLC* has shown up-regulated in precocious trifoliate orange. One possible reason may be that the regulatory mechanism of *CiFLC* was different between *Arabidopsis* and woody plants. In citrus, the expression profile of *CiFLC* showed up-regulation during the winter, followed by a decrease in the spring and summer. This kind of cycling is contrary to the pattern observed in *Arabidopsis* [29]. In addition, the relative expression level of the remaining genes was decreased sharply at higher concentrations of 5-AzaC indicating that they may perform a similar mechanism between citrus and *Arabidopsis*. In *Arabidopsis*, the extent of advancement of flowering time is dependent on 5-AzaC concentration, 250 µm being optimum. Higher concentrations of 5-AzaC were inhibitory to bolting and flower development was probably due to the nonspecific toxic effects of 5-AzaC [24].

To examine the differences in the DNA methylation status of five flowering related genes during phase change process, the full-length (including promoter) sequence of these genes was isolated. However, only one CpG Island was identified in *CiLFY* gene by bioinformatics analysis, the result indicated that the *CiLFY* may be regulated directly by DNA methylation. *LFY* is a transcription factor that affects not only inflorescence initiation but also floral organ determination in annual and woody plants [25], [32], [37]. Thus, *CiLFY* expression was analyzed at juvenile and adult stages. The expression pattern was closely correlated with floral induction, inflorescence development and flowering (Figure 3), suggesting that the gene may play a critical role in the flowering process of precocious trifoliate orange. Because precocious trifoliate orange was a woody perennial plant, it was difficult to observe a spatial gene expression pattern in the whole plant throughout its life cycle. Therefore, to examine the spatial expression patterns of *CiLFY*, we generated transgenic *Arabidopsis* with the *GUS* reporter gene driven by the regulatory sequences of putative promoters of *CiLFY*. According to our data for stable expression analysis, GUS expression in the transgenic *Arabidopsis* was consistent with the result of real-time PCR. These results indicated that transgenic *Arabidopsis* could reliably reflect the temporal expression of *CiLFY* in precocious trifoliate orange. Deletion analysis of the *CiLFY* promoter was also performed for determining the function of the *cis*-acting elements. The Del 1 and Del 4 had the higher activity among all the fragments in transgenic *Arabidopsis* because of the presence of inhibitor and enhancer in this promoter, suggesting that these sequences were important for the regulation of *CiLFY* promoter activity in citrus. Previous studies have reported that GAs affect plant flowering through a pathway that controls *LFY* transcription. In this study, GUS fluorescence assays has reflected the visible increase in the gene expression with GA_3_ treatment, but there was no significant difference between the different promoter deletion lines in response to GA_3_. Our finding explain that the GA_3_ regulatory sequence for inducting the gene expression was located somewhere between the −634/+1 fragment of the *CiLFY* promoter. The above fragment consists of only one GA_3_ induction *cis*-elements: PYRIMIDINEBOXHVEPB1 in −177/−169 region. Therefore, we speculated that the GA_3_ inducting *cis*-elements might be the main reason for *CiLFY* promoter response GA_3_. However, further studies were required because this was a preliminary, inconclusive deduction on our part.

We have performed the bisulfite sequencing in GpC island of *CiLFY* gene to examine whether the level of DNA methylation will alter during the phase change process or not. There was a decrease in level of DNA methylation at adult stage as compared with juvenile stage. In *Arabidopsis DDM1* mutants [60] expressing METI in antisense orientation [61], the decrease in DNA methylation affected the duration of both juvenile and vegetative phases and induced flowering [62]. Hence, METases must have a role in regulating developmental phase change [62], [63]. These findings indicated that *METases* might regulate *CiLFY* expression in precocious trifoliate orange. Previous report proclaimed that methylation was not detected in the *LFY* of *Arabidopsis* [44]. The difference of methylation and demethylation during regulation of transition development between annual and perennial plants might be correlated with their meristem determination. It has been reported that the molecular difference between perennials and annuals may be rather small, and a change between these life strategies might not require major genetic innovations [64]. In terms of floral process, floral evocation implies epigenetic reprogramming that shifts from vegetative to reproductive growth pattern. The decrease in methylation is related to gene activation and changes in morphology pathways [65], as well as being connected to the process of floral induction [24], [66].

## Conclusions

Epigenetic control, and specifically DNA methylation, plays an essential role in regulating the timing of precocious trifoliate orange flowering. Our findings also suggested that instead of all, only certain key genes were regulated directly by the DNA methylation by depending on related phenomena and species. According to the analyses of a series of truncated *CiLFY* promoter constructs, the present research has also demonstrated the importance of various regions of the *CiLFY* promoter for the regulation of citrus flowering. The “PYRIMIDINEBOXHVEPB1” motif was a critical element that determines GA_3_ induction of the *CiLFY* promoter. The reporter constructs described here will also provide a useful means of further analyses of *CiLFY* regulation, with a view to making comparisons with other promoters of flowering genes responsive to GA_3_, and developmental cues. Further studies will be required to clarify the function of *CiLFY* by analyzing the transgenic trifoliate orange. For example with an RNAi (RNA interference) construct and by investigating their spatial and temporal expression patterns by *in situ* hybridization in order to develop the technology to control the flowering of trifoliate orange in the juvenile and adult phase.

## Supporting Information

Figure S1 Methylation status of *CiLFY* gene at juvenile and adult stages of precocious trifoliate orange. Probable sites for the three classes of methylation (CGN, CHG, and CHH) as well as actually methylated sites in all the samples were identified by the software and projected symbolically. Blocked symbols represent actual methylation, whereas unblocked ones represent potential sites. A: Line diagram for 5′-UTR and CG island in *CiLFY* DNA sequence, a, b and c present region 1–3 in CpG island of *CiLFY* gene, respectively; d and e present region 1–2 in 5′-UTR of *CiLFY* gene, respectively. B: CG island of *CiLFY* methlyation analysis, a, b and c presents region 1–3 in CG island, respectively; d and e presents region 1–2 in 5′-UTR sequence, respectively.
(TIF)

Table S1 Primers for genome walking, 5′ deletions of *CiLFY* promoter, real-time PCR and methylation analysis.
(XLS)

Table S2 *Cis*-elements in the *CiLFY* promoter predicted by database analysis. Different motifs and models were identified using PLACE, PlantCARE and Regsite Plant databases and the Genomatix suite software. Positions are relative to the transcriptional initiation site. The orientation of the motifs is indicated (+, forward; −, reverse). Nucleotides are indicated as follows: N for A, C, G or T; Y for C or T.
(XLS)
