# Supplementary figures and images for: Possible Involvement of Locus-Specific Methylation on Expression Regulation of LEAFY Homologous Gene (CiLFY) during Precocious Trifoliate Orange Phase Change Process
Source: PLoS One. 2014 Feb 11;9(2):e88558. doi: 10.1371/journal.pone.0088558 (PMC3921215; doi:10.1371/journal.pone.0088558)

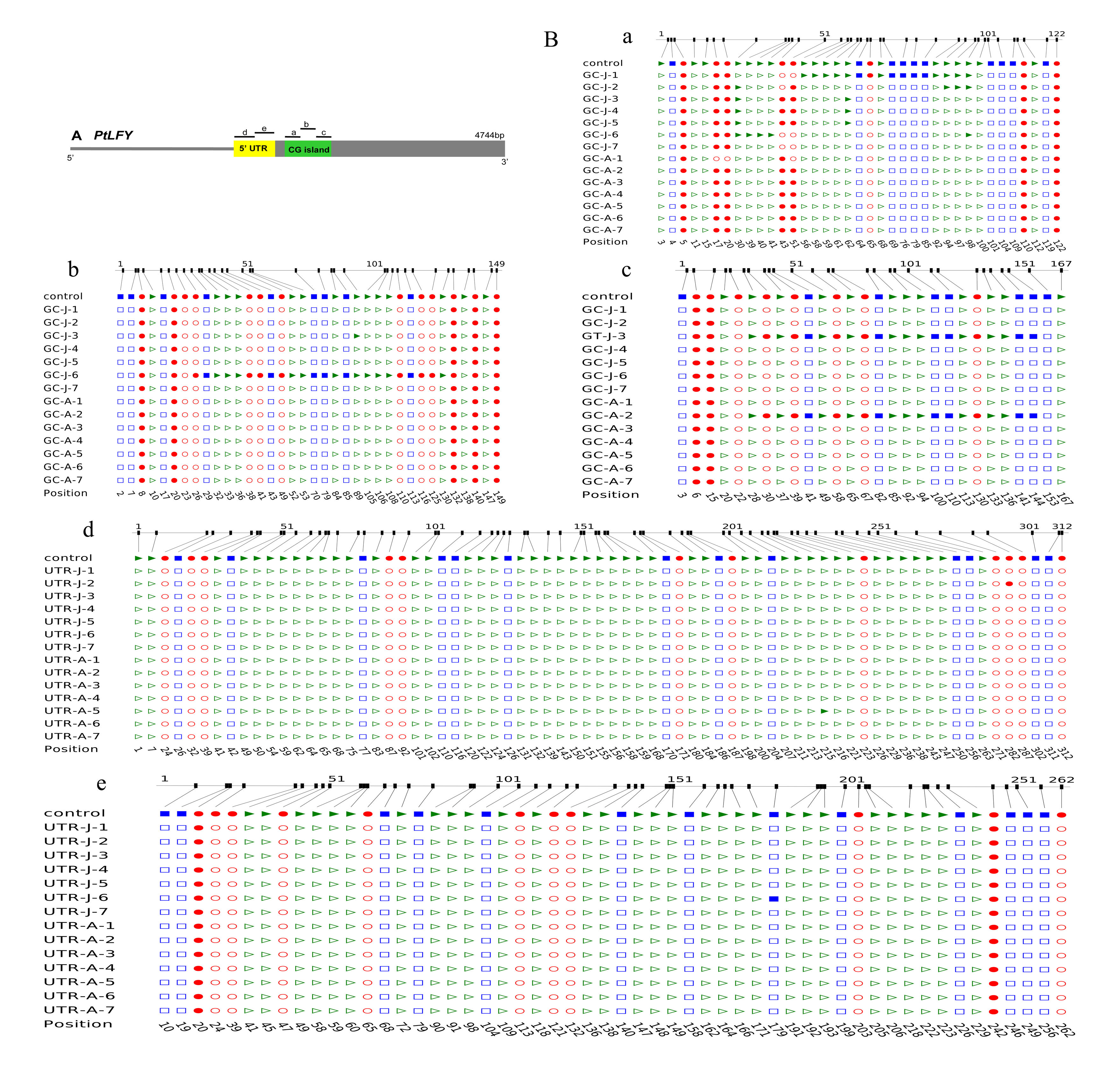

Supplement: Figure S1 — Methylation status of CiLFY gene at juvenile and adult stages of precocious trifoliate orange. Probable sites for the three classes of methylation (CGN, CHG, and CHH) as well as actually methylated sites in all the samples were identified by the software and projected symbolically. Blocked symbols represent actual methylation, whereas unblocked ones represent potential sites. A: Line diagram for 5′-UTR and CG island in CiLFY DNA sequence, a, b and c present region 1–3 in CpG island of CiLFY gene, respectively; d and e present region 1–2 in 5′-UTR of CiLFY gene, respectively. B: CG island of CiLFY methlyation analysis, a, b and c presents region 1–3 in CG island, respectively; d and e presents region 1–2 in 5′-UTR sequence, respectively. (TIF) [file pone.0088558.s001.tif]
